# Rapidly Deployed Phone-Based COVID-19 Telemedicine Clinic in Lebanon: A Retrospective Analysis

**DOI:** 10.5334/aogh.5463

**Published:** 2026-08-31

**Authors:** Rabih Soubra, Rim Taleb, Ibrahim Chehab, Yahya Makkieh, Alissar El Sibai, Issam Shaarani

**Affiliations:** 1 Faculty of Medicine, Beirut Arab University, Beirut, Lebanon; 2 IRADA, Federation of Businessmen for Support and Development, Beirut, Lebanon

**Keywords:** telemedicine, COVID-19, Lebanon, hotline, triage, supervision, academic-NGO partnership, health workforce

## Abstract

*Background:* In early 2021, Lebanon’s second COVID-19 wave coincided with financial collapse, health-workforce emigration, and the aftermath of the August 2020 Beirut port explosion, leaving hospitals saturated and many households unable to afford routine medical advice.

*Objectives:* To describe the design, utilization, disposition outcomes, supervisory escalation, and direct operational cost of a free phone-based telemedicine clinic rapidly deployed through an academic–nongovernmental organization (NGO) partnership, and to identify transferable lessons for other crisis-affected, resource-constrained settings.

*Methods:* Beirut Arab University Healthcare Center and the Federation of Businessmen for Support and Development (IRADA), a national NGO, launched a free 24-hour, 7-day-a-week phone-based telemedicine clinic staffed by 40 medical interns and residents using a standardized COVID-19 management protocol under a two-tier supervision model, with on-call family physicians reachable throughout each shift. WhatsApp voice calls were offered to provide free access, and missed calls were returned by the on-call responder. We retrospectively analyzed the de-identified records of all consultations managed during 14 weeks (February 2 to May 11, 2021); disposition categories were derived from the free-text recommendations using a rule-based classifier.

*Findings:* The service managed 2953 consultations from all Lebanese governorates. After clinical assessment and safety-netting, 90.1% of consultations were managed outside hospitals at the point of consultation, 7.8% were referred to the emergency department, and 2.0% to primary care. The on-call family physician was consulted in 6.6% of consultations, concentrated on the highest-acuity calls (16.5% of emergency department referrals). The overnight window carried 3.7% of consultations but a higher emergency department referral proportion (12.8%). Direct operational cost was USD11,032 (USD3.74 per consultation), excluding non-monetary university contributions.

*Conclusions:* An academic–NGO partnership, a standardized triage protocol, and tiered supervision can mobilize a trainee workforce safely, at low cost, and within days during a public health emergency.

## Introduction

In late 2019, COVID-19 emerged and rapidly escalated into a global health emergency, as declared by the World Health Organization (WHO) on January 30, 2020 [1]. Healthcare systems, in both high-income and low- and middle-income countries, suddenly found themselves overwhelmed by unprecedented pressure as patient admissions increased near-exponentially, intensive care units got full, and healthcare workers faced shortages of protective equipment [2, 3]. Early epidemiological evidence revealed the need to urgently slow COVID-19 transmission, especially among healthcare workers, in order to try to “flatten the curve” [4]. This call aimed to distribute cases over larger periods of time by slowing the spread of the virus so that the number of new infections per day stays reasonable and that the capacity of the healthcare system could accommodate it [4].

Telemedicine aims to provide care for patients using technology in order to communicate and deliver healthcare services [5]. It is known to be a way to expand access to healthcare, particularly when resources are limited or in times of crisis [5]. Enabling remote consultations through voice or video calls helps overcome geographical barriers and minimizes exposure risk during infectious outbreaks [5], while also being cost-effective [5]. During emergencies such as pandemics [6] or disasters [7], telemedicine can ensure uninterrupted access to primary care, triage support, chronic disease management, and mental health services. It is simply a flexible and adaptable solution in resilient health systems, making them capable of adapting to sudden surges in demand [5].

In Lebanon, the COVID-19 outbreak and the severe financial crisis created an urgent need to reduce unnecessary hospital visits [8]. At the same time, many individuals were unable to afford physician visits, were hesitant to seek healthcare due to fear of infection, or were faced with the fact that their physician had left the country [8–10]. Therefore, a low-cost remote solution was needed to maintain healthcare access during this crisis [8]. A Lebanese study conducted during the pandemic framed telemedicine as a way to support a struggling health system by reducing clinic visits, limiting exposure to infection, and saving time and transportation costs for patients and physicians, with perceived usefulness during the pandemic identified as an important determinant of physicians’ intention to use it [11]. The idea of a free medical hotline came up as a feasible model that reduces barriers to healthcare in Lebanon. Given the COVID-19 situation, economic collapse, fuel shortages, the aftermath of the Beirut blast, and increasing shortage of healthcare workers, this free hotline offered a practical solution to the needs of the population. It offered a low-cost, accessible method to help in triage, provide medical care, redirect patients to appropriate care when needed, and alleviate the pressure on the overwhelmed medical system. Here, we describe its design, utilization patterns, operational outcomes, and transferable lessons that may inform similar rapid-deployment efforts in other crisis-affected, resource-constrained settings.

### Lebanese context

When the first cases of COVID-19 emerged in Lebanon, the healthcare system was already struggling with financial challenges [9]. In a country that relies on a mix of public, private, and humanitarian resources to provide healthcare services, with wide variations in affordability among the population, COVID-19 was the tipping point of an already collapsing healthcare system [9]. The economic crisis that preceded COVID-19 emergence by a few months had its heavy toll on patients, providers, institutions, and access to resources [8–10]. The currency devaluation led to shortages of medical supplies, and a “brain drain” manifested by an estimated 40% of doctors and 30% of registered nurses leaving the country permanently or temporarily [8, 9]. When COVID-19 reached Lebanon in early 2020, hospitals were expected to quickly become overwhelmed with suspected and confirmed cases; however, this was not the case [9]. In fact, early on, only Rafik Hariri University Hospital was receiving COVID-19 patients. The first national lockdown occurred during March–May 2020, and it allowed other hospitals to prepare COVID-19 units [9]. The situation was under control, and relatively low case numbers and low positivity rates were maintained until the Beirut blast on August 4, 2020. The situation deteriorated further with the Alpha wave in January 2021, when ICU occupancy reached approximately 90% [9]. Later on, lockdowns were announced, mobility restrictions were enforced, and fear of infection pushed patients to delay seeking medical care [8, 9]. For many families, especially those facing financial distress, traditional healthcare visits became more challenging, creating an urgent need for alternative solutions [9, 10].

## Methods

### Study design

This article reports a retrospective, descriptive study of routinely collected service-delivery records from a phone-based telemedicine clinic. The clinic was implemented as a routine service-delivery initiative in response to a public health emergency, not as a research protocol; the research component of this work is the retrospective analysis of the de-identified records the service generated. In line with reporting guidance for quality-improvement and service-delivery initiatives (SQUIRE 2.0) [12], the methods first describe the program as it was implemented and then the data sources and analytic approach.

### Setting and partnership

The initiative was operated jointly by the Beirut Arab University Healthcare Center (BAUHC), a university-affiliated ambulatory care center established in 2018 to provide comprehensive ambulatory care and to host clinical rotations for medical interns and residents of the Beirut Arab University Faculty of Medicine, and IRADA, the Federation of Businessmen for Support and Development, a national NGO established in 2013 whose structure includes a Medical Care Committee of physicians and administrators with expertise in clinical and health-system management. The partnership was formalized in a Memorandum of Understanding signed in January 2021, which defined roles, decision-making, and accountability. A joint committee with representatives of both organizations oversaw planning and implementation, with weekly performance reporting from BAUHC to IRADA. BAUHC was responsible for clinical governance, staffing and scheduling of the supervised medical workforce, protocol development, training, data collection, performance monitoring, and the call center workspace; IRADA was responsible for fundraising, equipment procurement, promotional materials, community outreach, and coverage of operational costs, including hourly compensation for the medical workforce.

### Service model and access

The service operated as a 24-hour, 7-day-a-week phone-based telemedicine line accessible to any member of the public in Lebanon. The service was publicized through Arabic-language promotional posters disseminated on social media platforms, presenting the clinic as a free 24/7 COVID-19 phone clinic staffed by a medical team, listing the dedicated call numbers, and carrying the logos of both partner organizations. To minimize cost to callers in a deepening currency crisis, WhatsApp voice calls were offered as a free alternative to standard cellular calls. Any missed call was returned by the on-call responder, typically within one hour whenever a caller identifier was available, so callers did not bear the cost of repeated attempts.

### Workforce and supervision

At each shift, one to three medical interns or residents responded to calls under a two-tier supervision model: the responder conducted the structured triage, while an on-call supervising family physician was reachable throughout the shift to advise on complex cases. A total of 40 trainee physicians (29 interns and 11 residents) rotated through the service on weekly schedules built around fixed-length shifts, supported by a panel of eight family physicians. A small core team provided leadership: a medical director, a project administrative manager, a residents’ lead, a family physicians’ lead, and a public-relations coordinator.

### Clinical protocol

Recommendations followed a unified BAU COVID-19 management protocol developed by family physicians and updated continuously as international and national guidance evolved. After structured assessment of symptoms, exposure, comorbidities, vital signs reported by the caller, and red flags, callers were directed into one of four disposition pathways: (1) home management with explicit safety-netting; (2) general medical advice (isolation, infectivity, recovery, post-acute concerns, or vaccination questions); (3) referral to primary care or a relevant specialty; or (4) referral to the emergency department.

### Data collection and analysis

Each consultation was documented in a structured spreadsheet database capturing caller and patient identifiers, demographics, governorate of residence, household size, past medical history, medications, social history, recent contact with confirmed cases, vital signs reported by the caller, symptoms, COVID-19 testing status and result, the recommendation given, the identity of the responder, and whether the on-call supervisor had been consulted. Data were extracted weekly and stored on a password-protected device accessible only to authorized members of the team. For the present analysis, we exported all consultations recorded during the 14-week operational window and analyzed them in aggregate. The free-text recommendation field was recoded into the four disposition pathways using a rule-based classifier that identifies imperative emergency department referral language, home-medication dosing patterns, and primary care referral phrases, while excluding conditional safety-netting language. Direct operational expenses were summed across two cost categories (workforce reimbursement and equipment and materials) and divided by the number of consultations and the number of unique callers to obtain marginal direct costs. Equipment and workspace were modest: a Private Automatic Branch Exchange (PABX) system supporting three concurrent lines, three prepaid SIM-equipped cell lines with recharging cards sufficient for four months of continuous operation, three 3G wireless gateways, USB call recorders, corded landline handsets and headsets, and three i3-class laptops, all installed in a conference room at BAUHC.

### Ethics

Ethical approval for the retrospective analysis of de-identified service-delivery data was granted by the Institutional Review Board of Beirut Arab University on February 27, 2026 (approval letter issued March 4, 2026), under the protocol title “A Rapidly Deployed Phone-Based Telemedicine Service During COVID-19 in Lebanon: Design, Utilisation Patterns, and Operational Lessons.” Callers received care as part of routine service delivery rather than research; the analysis reported here used only retrospective, de-identified service records, and no personal identifiers were retained in the analytic dataset. The study was conducted in accordance with the Declaration of Helsinki.

## Results

### Utilization and caller profile

Between February 2 and May 11, 2021, across 99 calendar days of continuous operation, the service managed 2953 consultations (Table 1). Calls were placed by patients themselves in 59.4% of consultations and by a family member in 40.2%. Calls reached the service from every Lebanese governorate; Beirut and Mount Lebanon together contributed 76.2% of calls, and the remaining calls originated mainly from South Lebanon (7.8%), North Lebanon (6.3%), Bekaa (2.9%), and Nabatieh (2.8%). Patients had a median age of 41 years (interquartile range 30–55), and 53.0% were female; chronic comorbidities were reported in 49.7%, most commonly hypertension (20.3%) and diabetes mellitus (11.6%). The most common reason for contact was confirmed COVID-19 infection (69.3%), followed by suspected COVID-19 (13.0%), recovery from a recent episode (9.0%), general questions without infection or exposure (5.3%), and known exposure to a confirmed case (3.4%). Two-thirds of calls (67.4%) were first-time contacts; 84.9% of callers had symptoms at the time of call, and 81.1% had been tested for COVID-19. Among symptomatic callers, the symptom pattern was mixed respiratory and nonrespiratory in 55.5%, exclusively nonrespiratory in 31.6%, and exclusively respiratory in 11.9%.

**Table 1:** Caller characteristics, clinical presentation, and disposition outcomes of 2953 phone-based telemedicine consultations, Beirut Arab University Healthcare Center/IRADA COVID-19 Clinic, Lebanon, February 2–May 11, 2021.

DOMAIN	VARIABLE	NO. (%) OR MEDIAN (IQR)
Who placed the call	Patient self	1753 (59.4)
	Family member	1186 (40.2)
Call sequence	First-time call	1991 (67.4)
Sex	Female	1564 (53.0)
	Male	1389 (47.0)
Age, years	Median (IQR)	41 (30–55)
	Aged 60 years or older	584 (19.8)
Governorate	Beirut and Mount Lebanon	2249 (76.2)
	Other governorates	678 (23.0)
Comorbidity	Any chronic comorbidity	1469 (49.7)
	Hypertension	600 (20.3)
	Diabetes mellitus	344 (11.6)
Reason for call	Confirmed COVID-19	2046 (69.3)
	Suspected COVID-19	383 (13.0)
	Recovered from COVID-19	267 (9.0)
	Questions or concerns without infection or exposure	157 (5.3)
	Known exposure to a confirmed case	100 (3.4)
Symptoms at time of call	Any symptoms	2507 (84.9)
	Mixed respiratory and nonrespiratory (of symptomatic)	1391 (55.5)
	Nonrespiratory only (of symptomatic)	791 (31.6)
	Respiratory only (of symptomatic)	298 (11.9)
COVID-19 testing	Tested at time of call	2396 (81.1)
	Positive among tested	2204 (92.0)
Disposition	Home management with safety-netting	1736 (58.8)
	General medical advice	924 (31.3)
	Primary-care or specialty referral	60 (2.0)
	Emergency-department referral	230 (7.8)
Supervision	Escalated to on-call family physician	195 (6.6)

Abbreviation: IQR, interquartile range. Percentages are of the total cohort (*N* = 2953) unless otherwise stated; symptom-pattern rows use symptomatic callers (*n* = 2507) as denominator. Source: BAUHC/IRADA call center database.

### Disposition outcomes

After clinical assessment and safety-netting, 90.1% of consultations were managed outside hospitals at the point of consultation: 58.8% received home-management instructions with explicit safety-net advice, and 31.3% received general medical advice. The remaining 9.8% of consultations were directed to in-person care, comprising 2.0% to primary care or a relevant specialty and 7.8% to the emergency department.

### Temporal patterns

**Figure 1:**
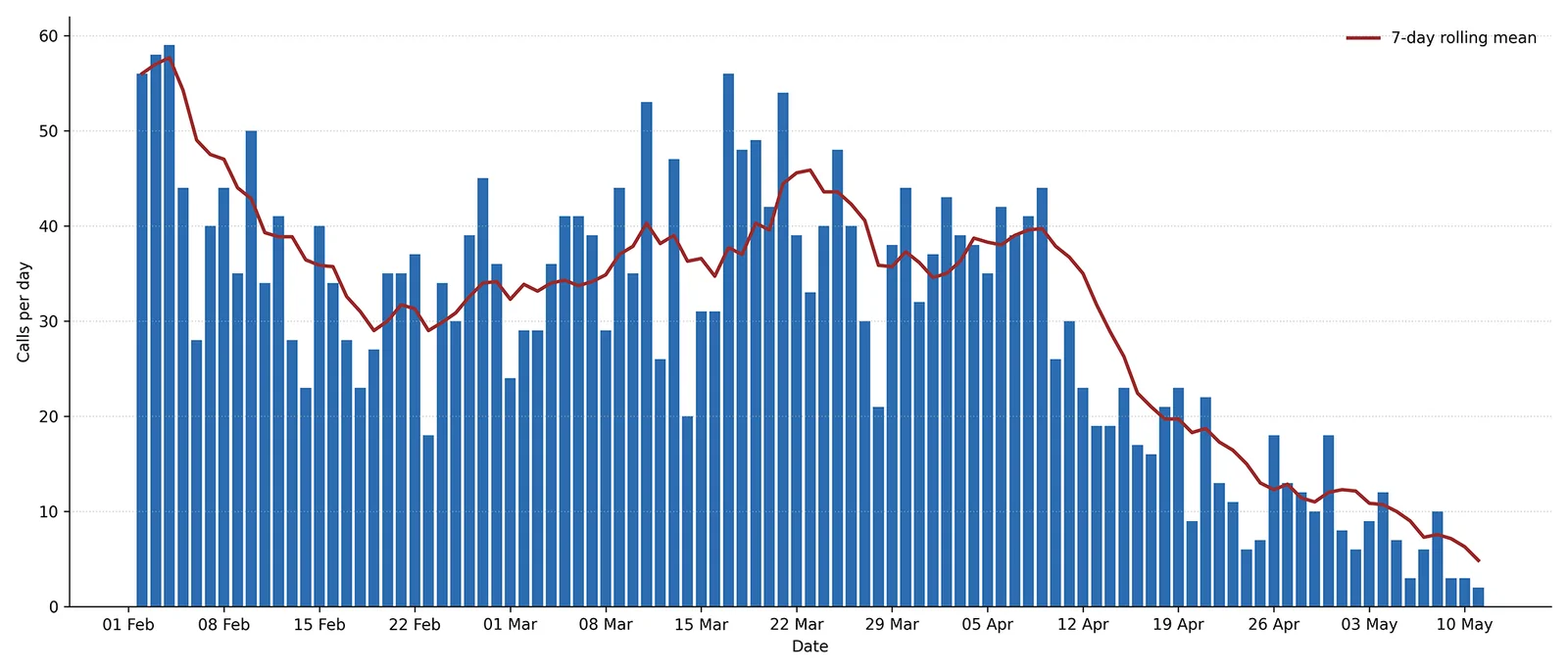
Daily Call Volume to the BAUHC/IRADA COVID-19 Phone Clinic, Lebanon, February 2–May 11, 2021. Daily counts of consultations across the 99-day operational window. Call volume rose sharply in the first days after launch, plateaued at approximately 30–45 consultations per day from mid-February through early April, and then declined steadily through April and into early May 2021 as the Alpha-variant wave receded and national case numbers fell.

**Figure 2:**
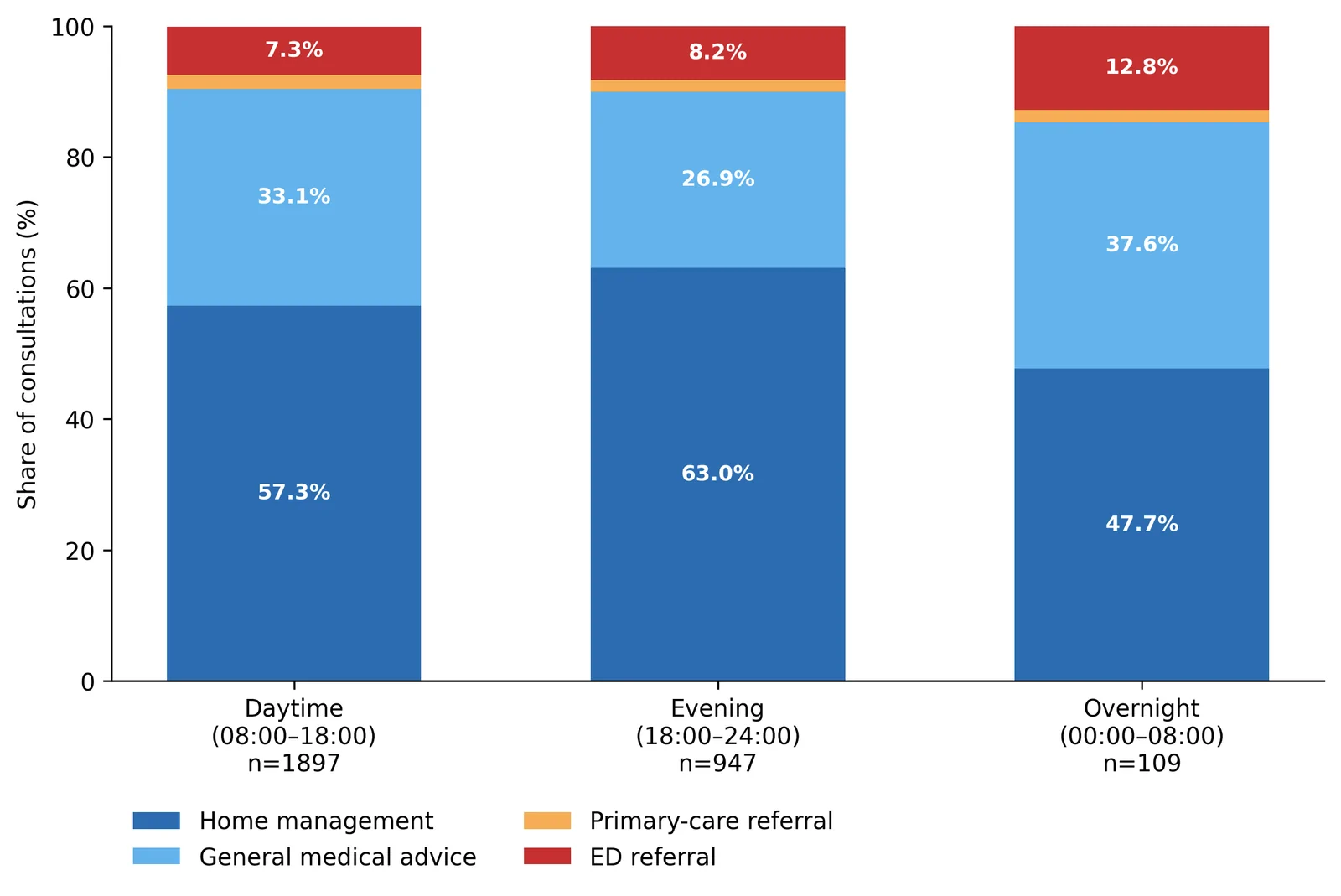
Disposition Outcomes by Time-of-Day Period, BAUHC/IRADA COVID-19 Phone Clinic, Lebanon, February 2–May 11, 2021. Distribution of the four disposition pathways across daytime (08:00–18:00), evening (18:00–24:00), and overnight (00:00–08:00) blocks. The overnight block had the highest emergency-department referral proportion (12.8%) despite the lowest absolute volume (109/2953 consultations, 3.7%).

Demand was sharply diurnal. The 12:00–18:00 window concentrated the highest volumes, with an absolute peak between 12:00 and 14:00 (Figure 1). The overnight window (00:00–08:00) carried only 3.7% of consultations, but the proportion of consultations resulting in an emergency-department referral was higher overnight (12.8%) than during the day (7.3%) or evening (8.2%), consistent with an acuity shift among callers contacting the service in the early hours. Correspondingly, the proportion of consultations managed outside hospitals was 90.4% during the day (08:00–18:00), 90.0% in the evening (18:00–24:00), and 85.3% overnight (Figure 2).

### Supervisor escalation

The on-call supervising family physician was consulted in 6.6% of all consultations. Escalation tracked acuity: rates were 16.5% for consultations referred to the emergency department, 5.5% for home-management consultations, and 6.2% for general-advice consultations, and were modestly higher in callers aged 60 years or older (7.2% versus 6.5%) and in those with at least one chronic comorbidity (7.4% versus 5.9%). Escalation was more frequent during the daytime block (7.9%) than during the evening (4.6%) or overnight (1.8%) periods, reflecting both supervisor availability during the day and the small absolute volume of overnight calls.

### Operating costs

Direct, out-of-pocket operational expenses for the initiative totaled USD11,032 over the 14-week period, comprising USD8356 (75.7%) for workforce reimbursement and USD2676 (24.3%) for equipment and materials (Table 2). Across the 2953 consultations, this corresponds to a marginal direct cost of approximately USD3.74 per consultation, or USD5.54 per unique caller (1991 unique callers, accounting for repeat consultations from the same caller). Workforce reimbursement accounted for most of the per-consultation cost (USD2.83), with equipment and materials adding a further USD0.91. Several costs were absorbed by Beirut Arab University as non-monetary contributions and are not included in this estimate: facility use, electricity, landline telephony, the time of the medical and administrative directors, and routine information-technology support. These figures therefore reflect the marginal direct cost of operating the service, not its full economic cost.

**Table 2:** Direct operational cost (USD) of the BAUHC/IRADA COVID-19 phone clinic over the 14-week window.

COST CATEGORY	TOTAL COST (USD)	SHARE OF DIRECT COST (%)	COST PER CONSULTATION (USD)
Workforce reimbursement (residents and interns)	8356	75.7	2.83
Equipment and materials	2676	24.3	0.91
Total direct cost	11,032	100.0	3.74
Cost per unique caller (*n* = 1991)			5.54

Raw expenditure: workforce USD8355.83, equipment USD2675.76 (total USD11,031.59); values rounded to the nearest USD1, with per-consultation costs to two decimals. Denominator is the 2953 consultations managed during the period; cost per unique caller uses the 1991 unique callers. Non-monetary contributions from Beirut Arab University (facility use, electricity, landline telephony, information-technology support, and director time) are not included.

## Discussion

### Principal findings

Through an academic–NGO partnership formalized in days rather than weeks, a free 24-hour phone-based telemedicine clinic in Lebanon managed 2953 consultations from every governorate during 14 weeks of operation, retained 9 in 10 callers outside hospitals at the point of consultation, concentrated supervisory escalation on the highest-acuity cases, and operated at a marginal direct cost of approximately USD3.74 per consultation. The diurnal pattern of demand and the higher emergency department referral proportion overnight have direct implications for staffing: the afternoon block warrants the densest staffing, while overnight provision retained a safety-net role even at low volume.

### Lessons for replication

Several features of the initiative appeared to contribute to its rapid deployment and sustained operation through the most severe weeks of Lebanon’s Alpha-variant wave. First, an academic–NGO partnership that aligned educational, clinical, and humanitarian mandates acted as a fast-track mechanism for service deployment. A Memorandum of Understanding, joint committee, weekly performance reporting, and explicit role allocation gave the partnership a clear governance backbone, while the NGO’s ability to mobilize financing outside formal procurement cycles allowed the service to launch within days rather than weeks.

Second, a standardized triage-and-management protocol with explicit escalation triggers can responsibly mobilize a junior workforce when trained physicians are scarce, provided that supervision is genuinely available throughout each shift and that the protocol embeds clear safety-netting and red-flag identification. The pattern of escalation we observed, concentrated on calls referred to the emergency department and other higher-acuity consultations, suggests that the supervision model functioned as intended.

Third, a phone-only service does not require costly digital infrastructure to be useful in a fragile setting. The deliberately modest technology footprint kept procurement timelines and operating costs low and avoided dependence on specialized digital-health platforms. It also made the service accessible to anyone with a cell phone, with or without internet connectivity. The low marginal cost of approximately USD3.74 per consultation, achieved partly by offering WhatsApp voice calls as a free option for callers and by university non-monetary contributions, suggests that the limiting factor for replication in many low- and middle-income settings is unlikely to be technology or finance, but rather governance, supervision capacity, and the political will to mobilize institutions across sectors.

### Limitations

This experience also has limitations. Outcomes were captured at the point of consultation and were not systematically followed up to verify clinical disposition decisions, and adverse events were not systematically tracked; the initial categorization of recommendations was performed by a clinical team member and recoded against a rule-based classifier prior to the present analysis, and full inter-rater agreement was not assessed; calls were recorded but not systematically audited for quality assurance, and no formal assessment of protocol adherence was performed; and the cost figures exclude non-monetary university contributions whose full economic value was not estimated. The experience is from a single country and a single 14-week window, and generalizability to other crises or longer time horizons should be assessed cautiously.

## Conclusions

In response to a confluence of crises, a free 24-hour, 7-day-a-week phone-based telemedicine clinic was deployed within days through an academic–NGO partnership and operated for 14 weeks at a marginal direct cost of approximately USD3.74 per consultation, retaining the majority of callers outside hospitals at the point of consultation and concentrating supervisory time on the highest-acuity cases. The model is replicable in concept and inexpensive in execution; what it requires is governance, a clinical protocol, a trained and supervised workforce, and a partnership that can move at the pace of a crisis.
